# A Random Forest approach to predict the spatial distribution of sediment pollution in an estuarine system

**DOI:** 10.1371/journal.pone.0179473

**Published:** 2017-07-24

**Authors:** Eric S. Walsh, Betty J. Kreakie, Mark G. Cantwell, Diane Nacci

**Affiliations:** U.S. Environmental Protection Agency, Office of Research and Development, National Health and Environmental Effects Research Laboratory, Atlantic Ecology Division, Narragansett, Rhode Island, United States of America; Universidade de Aveiro, PORTUGAL

## Abstract

Modeling the magnitude and distribution of sediment-bound pollutants in estuaries is often limited by incomplete knowledge of the site and inadequate sample density. To address these modeling limitations, a decision-support tool framework was conceived that predicts sediment contamination from the sub-estuary to broader estuary extent. For this study, a Random Forest (RF) model was implemented to predict the distribution of a model contaminant, triclosan (5-chloro-2-(2,4-dichlorophenoxy)phenol) (TCS), in Narragansett Bay, Rhode Island, USA. TCS is an unregulated contaminant used in many personal care products. The RF explanatory variables were associated with TCS transport and fate (proxies) and direct and indirect environmental entry. The continuous RF TCS concentration predictions were discretized into three levels of contamination (low, medium, and high) for three different quantile thresholds. The RF model explained 63% of the variance with a minimum number of variables. Total organic carbon (TOC) (transport and fate proxy) was a strong predictor of TCS contamination causing a mean squared error increase of 59% when compared to permutations of randomized values of TOC. Additionally, combined sewer overflow discharge (environmental entry) and sand (transport and fate proxy) were strong predictors. The discretization models identified a TCS area of greatest concern in the northern reach of Narragansett Bay (Providence River sub-estuary), which was validated with independent test samples. This decision-support tool performed well at the sub-estuary extent and provided the means to identify areas of concern and prioritize bay-wide sampling.

## Introduction

Estuaries are some of the most productive ecosystems in the world, providing critical habitat to many organisms in all life-stages. They are at the interface between the terrestrial and marine environments and are under sustained anthropogenic pressures from multiple effects such as habitat loss, exploitation of resources, and pollution [1]. The long-term presence of numerous classes of pollutants in estuary sediments have prompted concerns about the toxic effects of these contaminants on humans and wildlife [2–5]. Classes of contaminants include legacy pollutants such as PCBs (polychlorinated biphenyl), pesticides, and PAHs (polycyclic aromatic hydrocarbon), and those of emerging concern such as personal care products and pharmaceuticals that are distributed widely through wastewater treatment plant (WWTP) effluent [6,7]. Spatial models of contaminant distributions in sediments are becoming increasingly important tools for determining the environmental impact of these pollutants.

In aquatic systems, models of site-specific contaminant distribution are typically informed by field-collected empirical data that account for complex hydrological patterns. Many of the aquatic contaminant distribution models [8,9] are designed specifically for freshwater streams [10–14]; fewer models address the distribution of contaminants in estuarine systems [15]. The available estuarine models are non-spatial interpretations of point-process observations [5,16,17], in-situ evaluations and spatial modeling of point-process interpolations [7], or interpolated estuary geochemical properties with overlaid contaminant predictions [18]. These are all viable models; however, each of these approaches requires an adequate sampling density to evaluate distribution properties at broad scales. Full estuary data of this type are often not available because of the extensive resources necessary for their collection. In addition, regulatory agencies often focus sampling efforts in sub-estuaries or narrow areas of concern, e.g., point source discharge locations. Typical for this type of data, our spatially heterogeneous and sparse dataset of measured sediment concentrations of the contaminant triclosan (TCS), 5-chloro-2-(2,4-dichlorophenoxy)phenol, did not provide the necessary coverage to directly interpolate TCS levels throughout the entire estuary of interest. We addressed such data limitations by developing a decision-support tool that identifies contamination hot spots at the full estuary extent using limited sub-estuary data.

TCS is an antimicrobial compound found in a wide range of consumer goods, personal care products (e.g., toothpaste, soaps) and household textiles [19]. The predominant mode of entry to estuaries such as Narragansett Bay, RI, USA, is via domestic WWTPs [20–22], which typically remove between 58–99% of TCS before discharge [21,23–25]. Combined sewer overflow (CSO) systems [26] and on-site treatment systems also discharge TCS, but the organic contaminant discharge contributions from individual on-site treatment systems on estuarine systems is poorly understood [27]. TCS has a low solubility (4.62 mg L^-1^) in marine environments [7] and a half-life between 2–20 days [28]. However, it adsorbs readily to organic matter [29] and is not susceptible to degradation under anaerobic conditions. This persistence and its continuing discharge enhance its occurrence in marine sediments [30,31]. The presence of TCS in marine sediments and potential association with readily available environmental variables such as sediment composition and point source discharge made TCS a model contaminant to evaluate the decision-support tool.

The research goal was the development of a tool that uses the commonly used machine learning method Random Forest (RF) [32] and quantitative contamination data from sub-estuary sediments to identify contamination levels at the estuary extent. Ultimately, the result is a simple static representation of areas of greatest contamination concern, i.e., hotspots, which would inform more intense sampling initiatives. The tool should be easily transferable to other estuarine systems and implemented with readily available data. We define transferability as inclusion of variables that are readily available and easily derived. RF is a robust algorithm using an ensemble method of tree-based regressions to determine a response from a set of predictor variables. It does not rely on data distributional assumptions [33], which makes it ideally suited for modeling nonrandom sub-estuary data. Some of the training samples were intentionally collected in locations presumed to contain elevated levels of TCS, thus biasing the TCS concentrations towards the tails of the distribution. The objectives of this study were to: 1) develop a model of TCS contamination based on limited sub-estuary data within Narragansett Bay, and 2) use this model to identify areas of greatest TCS concern across the full extent of the Bay using point process data.

## Methods

### Study area

**Fig 1:**
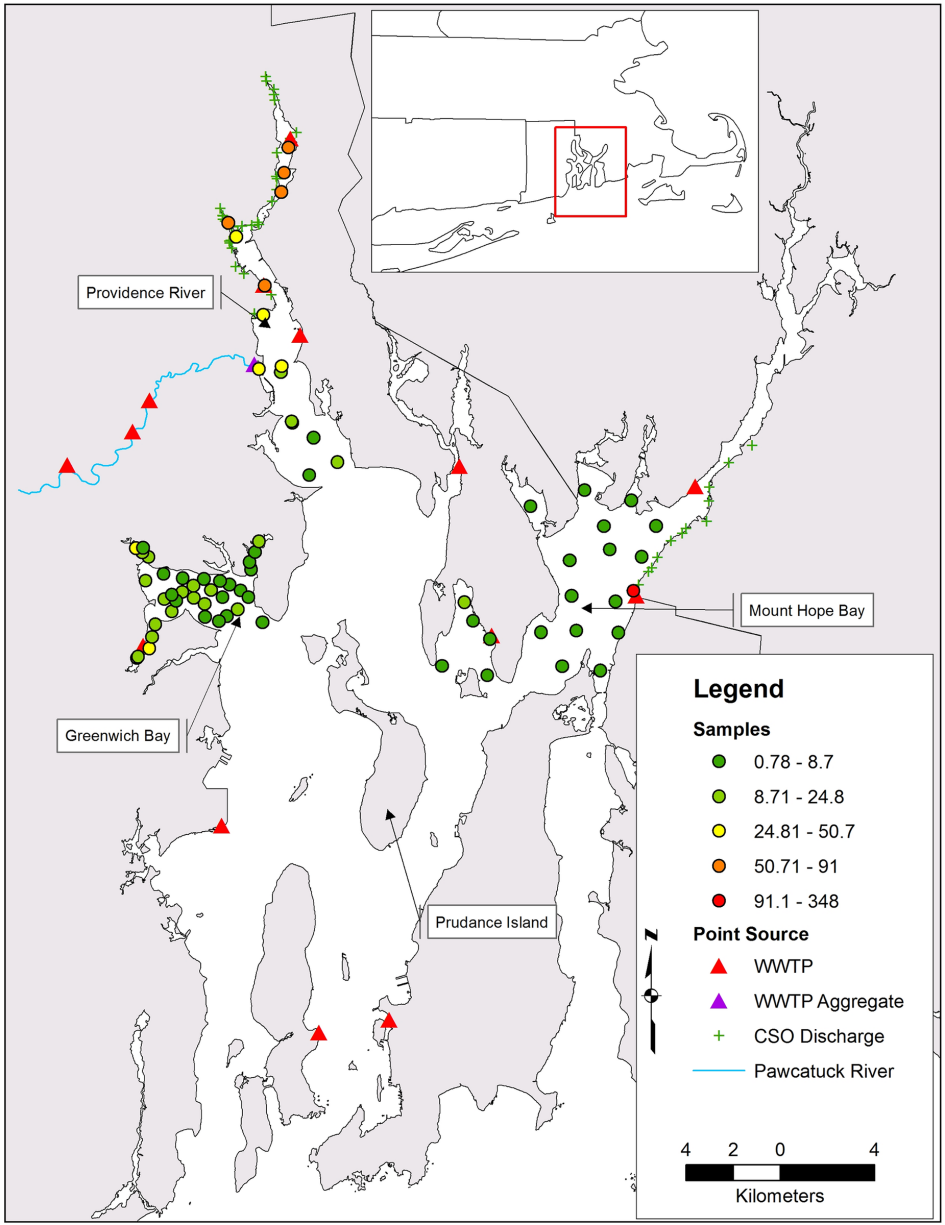
Distribution of triclosan training data and point source predictor variables in Narragansett Bay, RI. A sample’s color represents the observed sediment triclosan concentration (ng g −1).

The study focused on Narragansett Bay, an estuarine system extending north of Rhode Island Sound within Rhode Island and parts of Massachusetts. Narragansett Bay is surrounded by a watershed area of 4273 km^2^ with 60% in Massachusetts and 40% in Rhode Island and a total population size of 1.9 million [34]. There are three primary embayments or tidal rivers, which are referenced here using the term sub-estuary: the Providence River (PR), Mount Hope Bay (MHB), and Greenwich Bay (GB) (Fig 1). Approximately, 22% of the watershed population resides in the cities immediately surrounding the Bay north of Prudence Island with 7% in the immediate adjacent cities south of Prudence Island (Fig 1). The PR is a tidal river comprising the northern extent of Narragansett Bay (24 km^2^), and MHB (40.4 km^2^) is the east arm of Narragansett Bay at the terminus of the Taunton River and several smaller rivers. Combined, these two sub-estuaries sourced approximately 50% of the samples for the models presented here. There are two WWTPs that directly discharge treated effluent into the Providence River and four that indirectly discharge (three treatment plants discharge into the Pawtuxet River and one into the Seekonk River, all tributaries of the Providence River). Four of the WWTPs are closed systems discharging only treated effluent, while two plants are part of a CSO system. There are 93 CSO outfalls associated with the PR, of which 36 directly discharge and all are in the northern half. MHB opens to the south through the East Passage at Bristol Point. The Fall River WWTP and associated 19 CSO outfalls discharge into MHB. The Fall River treatment plant services 90,000 residents and storm water runoff from 20.2 km^2^ discharging 4.9 billion liters of treated effluent each year [35].

GB is a 12.2 km^2^ semi-enclosed sub-estuary within Narragansett Bay, Rhode Island. Water flow from Narragansett Bay is restricted into GB by a peninsula (Warwick Neck) creating a residence time of 8.8 days (Spaulding and Swanson, 2008). The East Greenwich Wastewater Treatment Plant, which discharges effluent into GB, services 6,000 residents producing an average flow rate of 3.0 X 10^6^ L d^-1^ [7].

### Explanatory variables

To achieve the study’s broader goal of developing a decision-support tool, explanatory variables (Table 1) were identified based on ease of acquisition, spatial extent, and empirical evidence supporting each variable’s hypothesized relationship with TCS. The variables corresponded to three broad categories associated with contamination: transport and fate, point source discharge, and non-point source discharge. Explicit modeling of estuarine hydrodynamics can be very complex [36], but it is important to effectively capture transport and fate dynamics. Total organic carbon (TOC), sediment composition, and bathymetry were included in the models to account for sorption properties of TCS to sediments and subsequent transport and fate but maintain model simplicity through implicit modeling. These variables were collected concurrent to the original TCS concentration data (see Model Development section). TOC was included, because the sorption properties of TCS results in a strong positive relationship between TOC and TCS concentration [7,22]. Sediment composition served as a hydrodynamic proxy, because sediment deposition is a function of flow speed and direction in Narragansett Bay [37–39], and the sorption of TCS to sediments and subsequent distribution are related [40]. Bathymetry was also included as a potential proxy for hydrodynamics because of its effects on sediment deposition [41,42].

**Table 1:** Explanatory variables for the triclosan Random Forest model with the rational for inclusion. Three different parameterizations (minimum, maximum, and average values) of the two point source variables were included in the models.

Variable	Process	Rational	Parameter	Value
total organic carbon	transport/fate	sorption	TOC	% TOC
surficial sediments	transport/fate	sorption; hydrodynamics proxy	sand (≥62.5 μm) mud (<62.5 μm)	% composition
bathymetry	transport/fate	hydrodynamics proxy	depth	bathymetry (ft)
point source	environmental			
combined sewer overflow	entry	point source	CSO_min,max,avg_	functional distance (m)
wastewater treatment plant			WWTP_min,max,avg_	normalized inverse functional distance weighted by permitted discharge volume
geographic location	environmental entry	anthropogenic proxy; non-point source	UTM Northing, Easting	meters East, meters North

Point source and anthropogenic non-point source discharges were included to account for environmental entry and spatial correlation of TCS. Direct environmental entry effects were parameterized based on distance to WWTP and CSO discharge locations; TCS concentrations were expected to be inversely related to distance [43] and strongly associated with WWTP discharge levels [7,26,44]. Three WWTPs discharge into Narragansett Bay indirectly via the Pawtuxet River (Fig 1). These plants were represented by one discharge location at the Pawtuxet River terminus. CSO discharge locations that did not directly discharge into Narragansett Bay were excluded, because they extended into tributary waters beyond the study’s extent, and a single aggregate point would not have accurately represented discharges. There is a positive relationship between total effluent discharge and total TCS discharge [7,45]. However, there was no available direct measure of TCS load for all point sources for this study. To account for presumed heterogeneous loads, the distances to WWTPs were weighted by the average daily discharge volume permitted [46] (the discharge volumes for the two Massachusetts WWTPs were based on the EPA permitted average daily flow). We calculated the weights using the following general calculation: *normalized inverse distance* among all WWTP multiplied by the *normalized discharge volume* among all WWTP, rescaled between one and two. As distance to discharge location decreased and daily discharge increased, the rescaled values increased (approached four). This transformation standardized the effect of WWTPs on a random location, because using just the raw distance and discharge volumes would misrepresent the effect of a WWTP on a random location. In addition, regression trees are invariant to transformations of explanatory variables because only the rank order determines a split at a node [47]. The CSO distances were not inverted or rescaled, because discharge volume information was unavailable. Finally, each measured TCS data point was parameterized with the minimum, maximum, and average cost-distance to WWTP and CSO discharge locations (S1 and S2 Figs), because there was no *a priori* assumption of parameter inclusion.

We assumed non-point source entry was predominantly related to on-site treatment systems [27]. To maintain model simplicity, non-point sources were modeled using each sample’s UTM (Universal Transverse Mercator) coordinates (Zone 19) as an “anthropogenic influence” proxy. A UTM position is an easting and northing planar coordinate. Narragansett Bay has an decreasing human population gradient from north to south with a corresponding shift from point source pollution (WWTPs and CSOs) to non-point source pollution (onsite waste water treatment) that has observable effects [48,49]. We hypothesized TCS concentrations may generally decrease from north to south due to this population gradient effect on non-point source pollution.

All distance measures for direct point sources were calculated using cost-based distances. The alternative Euclidean distance assumes equal accessibility to all areas, which is violated in an estuary. In an estuary, landmass edge represents an impenetrable “hard” barrier to contaminant movements. Therefore, using Euclidean distance to evaluate the spatial relationship between locations in an estuary would result in potential model bias [50]. Cost-based distance “as the fish swims”, an alternative to Euclidean, provides a more functional estimate of distance [51]. Functional distance models are based on the rasterization of the estuary and a calculation of the total “cost” of moving between two points as a function of traversing raster cells. The least cost path is then considered the “effective distance” [52]. Effective distances were calculated using a 15 m resolution grid of Narragansett Bay. The cells intersecting landmasses were removed, and each estuarine raster cell was coded as the maximum cost of traversing the cell, i.e., 15. This process resulted in cost-surface for each WWTP and CSO discharge location representing the cost-distance from the respective discharge location to every location in the Bay (S1 and S2 Figs). Cost surfaces were calculated using the algorithm r.cost in GRASS [53], an open source GIS program.

### Model development

A RF regression model was used to predict TCS concentrations based on a suite of predictor variables. The RF was implemented in R (v. 3.2.2) [54] using the *randomForest* package [55]. The algorithm is optimized via three parameters: *ntree-* the number of trees grown from a bootstrapped sample (*ntree* = 4000); *mtry*-the number of predictors randomly tested at each node (*mtry* = 9 was determined via randomForest’s *tuneRF* algorithm); and *nodesize*-the minimal size of the terminal node (*nodesize* = 1); see Reference [55] for more details. The RF training dataset was comprised of georeferenced samples of TCS concentration measured as ng g^−1^ of dry sediment (0.78 ≤ x ≤ 348, $\bar{x}=\;18.43$, σ = 46.85), surficial sediment percent composition, TOC, and bathymetry throughout Narragansett Bay. The data were collected in the spring of 2010 (n = 36) and April-October 2012 (n = 22) with a majority of the samples originating from GB alone (Fig 1). For a complete review of sampling methods, see Reference [6]. RF is useful for modeling very small sample sizes as ‘big data’ sets are not required to successfully employ RF [56]. The response variable was log-transformed to improve model fit, because for regression trees non-constant variation of the residuals gives greater weight to higher variance data [47]. Transformation of environmental predictors is unnecessary as RF can successfully handle non-normality [57].

Random Forest produces an unbiased error estimate via bootstrapping; this enables model validation without a secondary independent dataset [32,58]. The process proceeds as follows: for the *k*^th^ regression tree, a training set is sampled with replacement from the entire dataset leaving one-third of the cases out, i.e., out-of-bag (OOB) data. Each case (*i*) left out of the *k*^th^ tree’s construction is evaluated down the *k*^th^ tree creating a predicted test set for the *i*^*th*^ case from all the times *i* was OOB. For each *i*, an average predicted value is assigned from *i*’s test set resulting in a mean squared error calculation (MSE_oob_) [32] as follows:

$$MSE_{oob}=\frac{1}{n}\sum_{i=1}^{n}{\left(y_{i}-{\bar{\hat{y}}}_{iOOB}\right)}^{2}$$

The percent variance explained by the model is 1- MSE_oob_/ σ^2^(y), which is reported here as a model validity metric.

While RF can handle confounding variables [59], we sought a final prediction model that optimized variance using a parsimonious set of variables for prediction. In this regard, the package *VSURF* [60] was implemented within R to select a minimum set of explanatory variables from the full RF model (i.e., all variables) that were highly related to the observed TCS concentrations. The method proceeds by first calculating variable importance scores and eliminating variables of small importance based on importance ranks (descending order). Variable importance is measured by mean squared error of a variable *p*, which is the averaged increase in prediction error among all regression trees when the OOB data for variable *p* is randomly permutated. Thus, if variable *p* is important there will be an observed increase in prediction error after the *k*^*th*^ tree’s OOB data with the randomly permuted variable *p* is tested down the *k*^*th*^ tree. The second step of the variable selection procedure is to construct nested models of the ranked variables from the first step; the variables resulting in the lowest model OOB error are selected as the core explanatory variables, i.e., interpretation model. This set is further truncated to eliminate all but one of any correlated variables resulting in the most parsimonious prediction model. This parsimonious model was used to produce Bay wide predictions of TCS.

### Model predictions

The final static representations of TCS contamination in Narragansett Bay were predicted TCS concentrations using a point process dataset that were then categorized into three ordinal classes of “high”, “medium”, and “low”. The point process dataset was an aggregate of three sediment composition datasets: the U.S. Environmental Protection Agency published [61] (n = 69) and unpublished data (n = 14) (provided in repository), and Reference [62] (n = 363), with corresponding TOC values; grain size categories were consistent with the training data. The remaining predictor variable values were assigned to each point. Bathymetry data were extracted from a 15.24 m (50 ft) resolution bathymetry grid of Narragansett Bay [63] and a vector dataset converted to a 30 m resolution raster for the northeast section of MHB within Massachusetts [64]. This latter dataset was used for a minimal spatial extent and had a minimal effect on the results. Point and non-point source values were assigned the respective WWTP and CSO parameterizations and geographic coordinates. To facilitate identification of areas of greatest contamination concern, the logarithmic scale TCS predictions were discretized using data quantiles. Three different quantile threshold models (A, B and C) were developed. Specifically, model A represented an equal distribution of the TCS concentration predictions among the contamination classes (i.e., 33% of data in each class). Models B and C subsequently narrowed the range of TCS concentration predictions in the “low” and “high” classes and broadened the range of concentrations representing the “medium” contamination class. Model B designated 25% of data into the low and high classes, while 50% is lumped in the medium class. Model C restricts the low and high classes further to 12.5% of data. Therefore, between models A to C, the “high” class represented an increasingly higher average level of contamination. A dataset of TCS concentrations (n = 11) was used to validate predictions in the Providence River sub-estuary. These data were withheld from model development, because they lacked TOC values. The result of the quantile models was three static representations of the Narragansett Bay estuary indicating areas of contamination concern.

## Results and discussion

### Model performance

**Fig 2:**
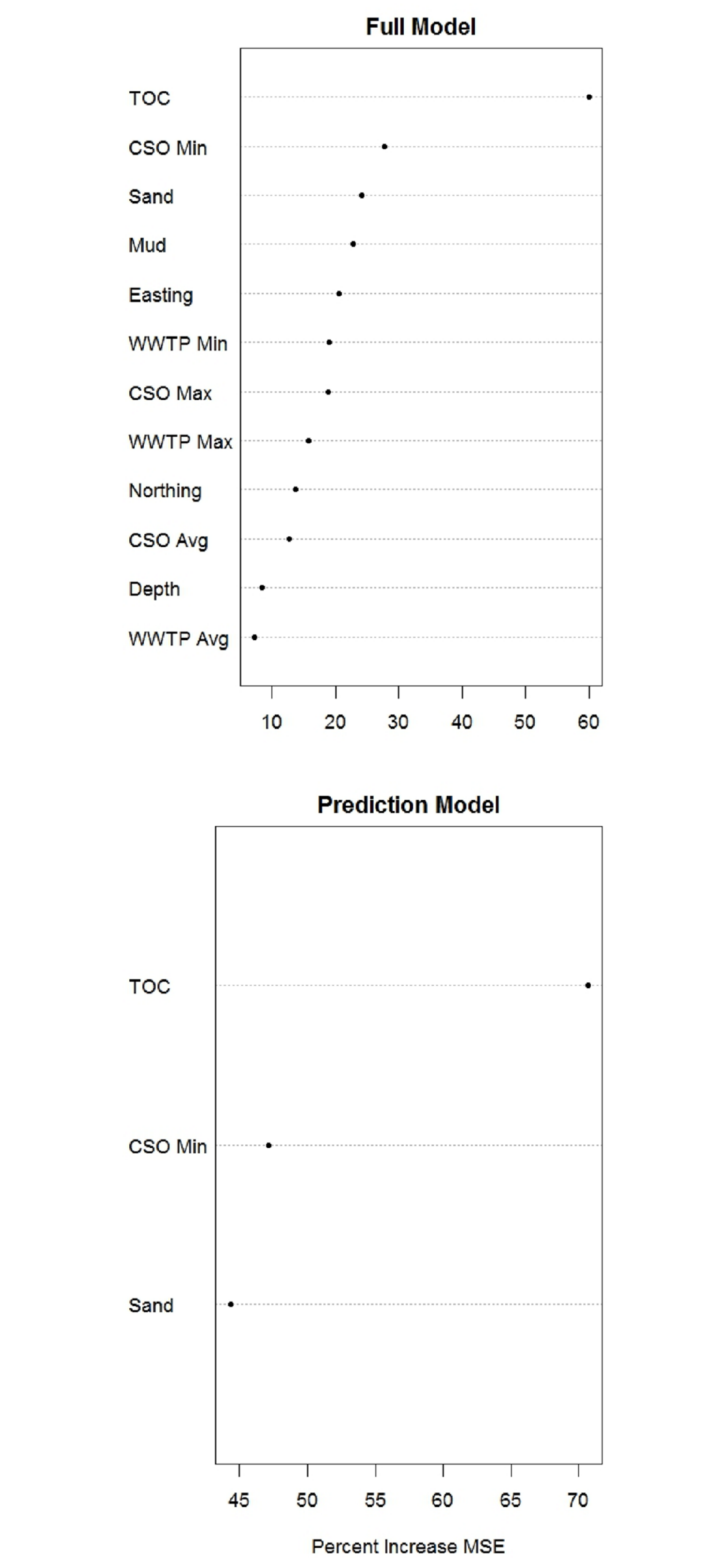
Random Forest variable importance ranks for the full and prediction model, which the latter was used to predict triclosan contamination in Narragansett Bay, Rhode Island. Percent increase in MSE (mean squared error) is a measure of variable importance; the closer to zero the less important the variable is for predicting. CSO, combined sewer overflow; Mud, sand/silt composition; Northing and Easting, geographic coordinates; TOC, total organic carbon; WWTP, wastewater treatment plant.

The full and prediction models performed well with a percent variance explained of 63% and 68.5%, respectively and a mean of squared residuals of 0.49 and 0.42, respectively. The interpretation model and prediction models contained the same variables (TOC, CSO_min_, and Sand). Based on the percent increase of mean squared error, TOC was the most important predictor of TCS with a mean squared error increase of 59% (full model) compared to permutations of randomized values of TOC (Fig 2). Previous research on TCS in GB indicates a strong relationship between TOC and sediment TCS concentrations (*r*^*2*^ = 0.89) [7] supporting the variable’s high rank. Sand was the only hydrodynamic proxy and additional sorption variable that was a strong predictor of TCS. Mud was a moderate predictor because of the high potential for TCS to sorb to organic matter and fine particles [30]. However, sand and mud were highly correlated composite variables and mud was not retained in the prediction model. Based on the RF partial dependency plots, the relationship between the composition of sand and TCS concentrations in these models was negative. Therefore, as percent composition of mud increased, sand decreased, and TCS concentration increased.

Point source discharges were strong to moderate predictors of TCS with a greater importance rank of the CSO parameter in relation to the WWTP (Fig 2). There are two hypotheses for this result: 1) the discharges of CSOs compared with WWTPs have a greater effect on TCS concentrations in Narragansett Bay; 2) multicollinearity is arising between variables due to sample bias or true collinearity because of underlying processes. It is possible the discharges from CSOs had a greater effect on TCS contamination compared to WWTP discharges because of the filtering of TCS by WWTPs. A majority of the training samples (62% of n) were from GB, which is subjected to TCS from advective processes and a single WWTP that filters 63–89% of the influent TCS [7] (Fig 1). The filtering efficiency of the other WWTPs contributing to Narragansett Bay is unknown, however an estimated 96% (activated sludge) and 58–86% (trickling filter) of TCS is removed from WWTP systems [44]. This explanation may not be probable based on loading potentials. Though, the total TCS discharge from WWTPs and CSOs in Narragansett Bay is unknown, estimates of total annual loadings of TCS from WWTP to U.S. waterways are 50–56% (activated sludge) and 39–47% (trickling filter) [26]. CSO discharges account for a relatively small amount (3–5%) of the total annual TCS load to U.S. waterways [26]. It is possible that the load from CSOs is greater because of the filtering effect of WWTPs, but considering the expected load from these respective point sources, it is not probable.

Alternatively, the higher rank of CSO point sources compared to WWTPs could be due to multicollinearity, sample bias, or anthropogenic population density effects. Many of the training samples were nonrandom and intentionally collected in locations presumed to contain elevated levels of TCS. WWTP discharges were located throughout the Bay; however, the samples were disproportionately distributed in the northern half of the Bay, which contains WWTP discharges associated with CSO outfalls (Fig 1) and is the area of greatest anthropogenic population density (see below). The location of WWTP discharges are not necessarily correlated to CSO outfalls or population centers. However, the converse is true, and this correlation is possibly affecting variable importance, because RF variable importance estimates maybe overestimated for highly correlated variables [65]. Two of the WWTP parameters were ranked as moderate predictors in the full variable model and were retained in the first iteration of the *VSURF* procedure, which makes a general selection for important variables (positive variable importance values). The subsequent iterations of the variable selection process address multicollinearity by retaining one of several highly correlated variables [66]. Whether CSOs ranked higher because of sample bias or proxy effects did not inhibit our objectives. Our objective was to create the best predictive surface based on already established variable relationships with triclosan and not parse new variables associated with triclosan contamination in the Bay. In regards to these influences on model prediction, Reference [63] noted, collinearity is a problem when “a model is trained on data from one region or time, and predicted to another with a different or unknown structure of collinearity”. This problem is not realized in this system, since the predictions were being made across the same modeling space within a similar period.

There was little support for the specific anthropogenic non-point source proxies in these models. The Northing parameter was expected to perform as a moderate to strong predictor of TCS concentrations and account for a potential north-south contamination gradient [67–69] similar to the human population density gradient in RI. The northern terminus of Narragansett Bay (Providence River) has a predominatly urban surrounding land use. The population density decreases and land use shifts to more residential in a southerly direction resulting in fewer point source discharge locations but increases in more onsite wastewater treatment systems (non-point sources). The Northing parameter failed to account for this effect in the models. There are several reasons for this result: non-point sources do not significantly affect TCS concentrations in Narraganset Bay, coordinates are a poor proxy, or the limited latitudinal distribution of samples is limiting the detection of non-point source effects.

### Contamination predictions

**Fig 3:**
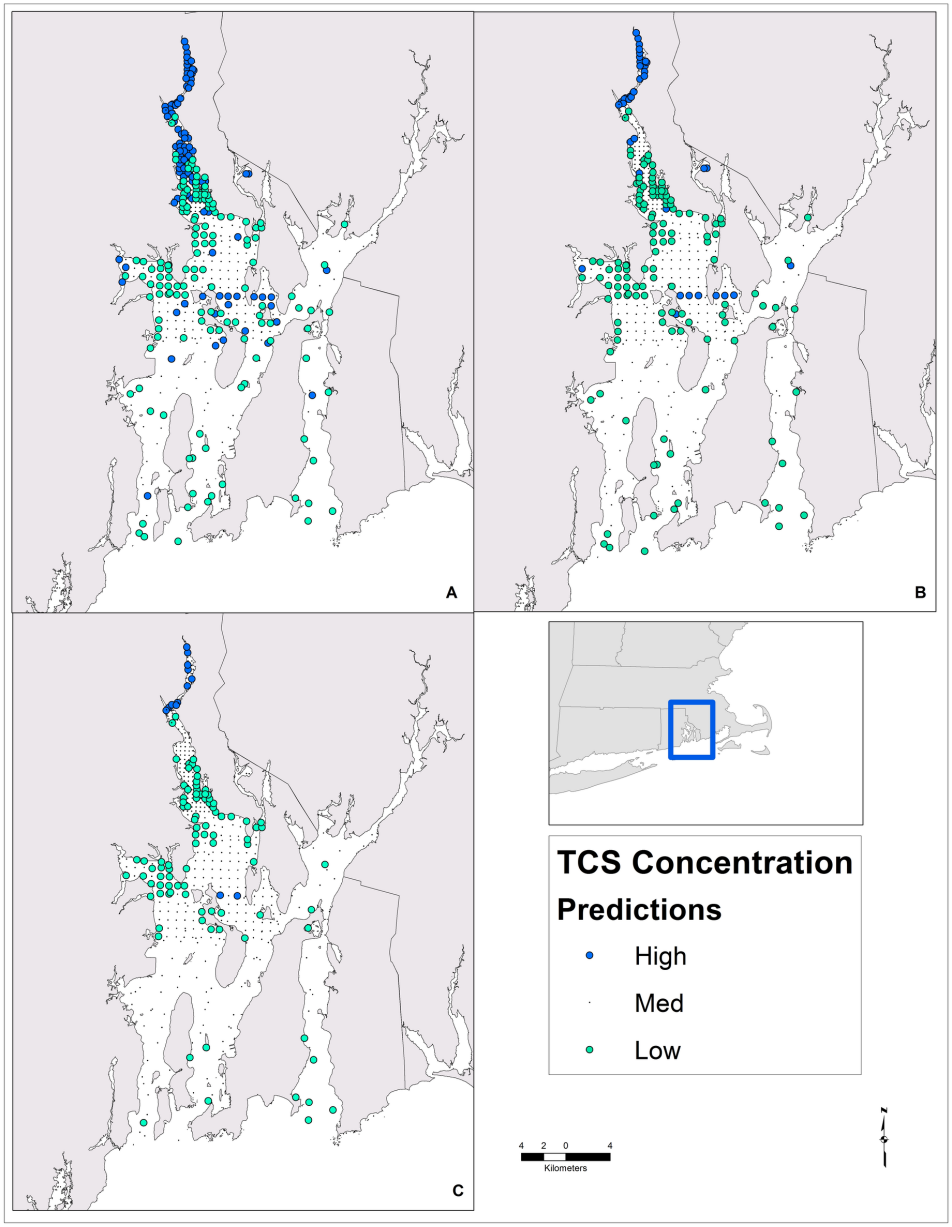
The predicted distribution of triclosan contamination based on three different quantile discretizations of a Random Forest regression model predictions. Maps A, B, and C represent the respective models (A, B, C). The medium contamination class points have been minimized to improve visualization of the low and high contamination classes.

**Fig 4:**
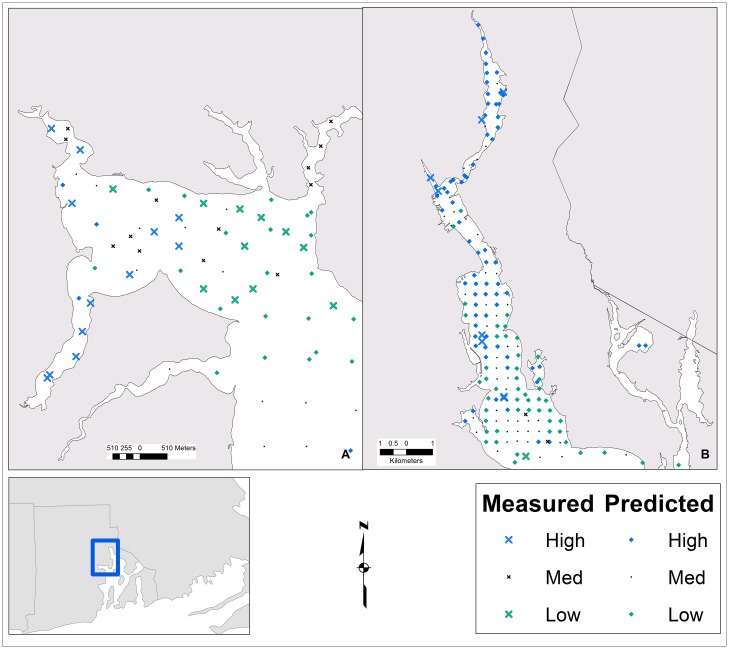
The distribution of model A predicted and measured triclosan contamination in the Greenwich Bay (A) and Providence River (B) sub-estuary of Narragansett Bay, Rhode Island.

The predicted TCS concentrations were binned into three ordinal levels of contamination using three different quantile thresholds. This resulted in the spatial distribution of contamination hotspots ranging from broad to narrow (Fig 3). Among all three models, the largest predicted TCS contamination hotspot was in the northern reach of the Providence River. This prediction is considered valid based on the spatial location of the hotspot, independent TCS measurements of the Providence River (Fig 4), and concordance with other distributions of contaminants within the Bay. The increased contamination potential in the PR seems likely, because it is subject to the direct and indirect discharge of 36 and 57 CSO outfalls, respectively and three direct WWTP discharges. Among the three threshold models (model A reported, Fig 4), the predicted PR hotspot is in general agreement with independent TCS measurements of the Providence River, which were withheld from the training data. The RF model predicted a north to south gradient of higher to lower concentrations in the PR, which was expected and is similar to the distributional patterns of other sediment bound contaminants (mercury and methyl mercury) reported by Reference [18]. In addition, the model predicted an overall north-south PR gradient even though the data that informed the Random Forest model contained a minimal number (four) of biased (all were in close proximity to WWTP discharges) samples from the Providence River (Fig 1).

The southeast and southwest sections of the Providence River and the mouth of Greenwich Bay were areas of predicted low contamination. We lacked an independent dataset for GB; however, for model A the low contamination predictions for the east section of GB were in agreement with the measured concentrations from the training samples (Fig 4). Like the PR prediction gradient, the GB contamination pattern of high to low (west to east) exhibited a similar pattern to mercury contamination [18]. There was an elevated contamination prediction in the central part of Narragansett Bay near Prudence Island and northeast of Prudence Island. Farther south throughout the Bay, the models predicted a predominantly low to medium mix of TCS contamination. The Bay processes contributing to the predicted increased contamination around Prudence Island is unknown; however, this pattern is similar to the mercury distribution reported by Reference [18]. The concentration patterns and gradients of other contaminants observed in Narragansett Bay have been attributed to hydrodynamics. The patterns predicted here are not in complete agreement with the west to east concentration gradient (high to low, respectively) presented by Reference [65]. In the full model, the Easting parameter was a moderate predictor of TCS but was not a core variable in the predictor model. The Bay wide north to south (high to low) TCS contamination gradient predicted by the RF model was similar to gradients of nitrogen isotopes [68] and mercury contamination [18]. Generally, the patterns predicted by the RF model have been observed in other contaminants and models, though there is less certainty about TCS contamination surrounding the Prudence Island region and south compared to the PR and GB sub-estuaries. Together, these unexpected hotspots may reflect depositional areas for distantly-contaminated sediments originating, for example, from more northern urban areas.

Evaluating contamination based on classes instead of raw concentration values was beneficial, because it shifts interpretation from fine scale predictions of concentrations to broad scale patterns of concentration ranges or levels. Contamination classes are useful for identifying areas of most concern, especially if toxicity is unknown. Specifically, broad quantiles (model A) simplify identification of areas of greatest contamination concern when highly toxic contaminants (i.e., toxic at low concentrations) with high ecological or human health risk are modeled. Compared to varied quantile thresholds, there are multiple alternate discretization strategies (equal interval, moment matching) that could be implemented. A strategy based on toxicity thresholds or other known protection limits, such as contaminant-specific sediment quality guidelines could link predictions with expected ecological effects. The strategy chosen should relate to the contaminant’s toxicity and purpose for evaluating its environmental effects. Overall, representing contamination based on broad concentration classes instead of raw concentration values improves interpretation and visualization of predictions.

### Model application

The transferability of this modeling approach for TCS contamination to other estuaries is possible. Transferability as defined here refers to the inclusion or exclusion of certain explanatory variables when applied to other appropriate estuarine systems and not the specific model applied here. TOC, WWTP, CSO, and sediment composition are likely important predictors of TCS contamination in other estuarine systems and should be included. These factors are related to TCS’s entry, transport, and fate within an estuarine environment and are not necessarily intrinsic to the Narragansett estuary. However, the degree to which they affect the distribution of contaminants is likely relative to the estuary being studied. Other variables such as the geographic coordinates, which were proxies for non-point source effects and hydrodynamics, were study specific and are not necessarily transferable to other systems. However, population density, population gradients, or hydrodynamics are likely important in other estuarine systems and inclusion of appropriate spatial metrics or proxies for them would benefit model development. Population effects could be assigned to samples using the population density within a fixed or nested buffer around each sample. Transferability could be evaluated using existing TCS data in other estuarine systems and by acquiring the variables evaluated here and others necessary to account for local processes. Finally, the size and inherent spatial relationships of the applicable estuary should be considered prior to application of this process.

## Conclusions

We presented one modeling component of a decision-support tool that predicted TCS contamination hotspots in an estuarine system. The model used Random Forest and minimal nonrandom sub-estuary contamination data to make the full estuary extent predictions. The TCS contamination predictions and model selected explanatory variables were in general agreement with expectations, independent data, and published contaminant distributions. However, certain explanatory variables such as WWTP discharges did not perform as expected probably because of sampling bias. This resulted in model uncertainty in extrapolations beyond the spatial extent of the training data. Such bias is not unique among sub-estuarine datasets. To improve model interpretation and identification of contamination hotspots that could inform future sampling efforts, specific predicted concentrations were aggregated into broad qualitative bins of contamination concentration ranges. Quantile thresholds can be based on regulatory limits or known relationships between contaminant concentrations and ecological risk. The robustness of this decision-support tool also depends on the transferability to other estuarine systems. Elements of the TCS model that account for the attributes associated with the chemical’s entry into the environment and sorption behavior are likely applicable across systems. Thus, the model should be considered a viable tool for similar applications, but additional research is necessary to evaluate the model’s overall robustness when applied to different contaminants and the transferability of this process to other estuarine systems.

## Supporting information

S1 Fig This is an example of the rescaled and weighted cost surface representing the field’s point wastewater treatment plant, which had the highest permitted average daily flow volume (45 million gallons^-day^) in this model. (JPG)

S2 Fig This is an example of the rescaled and weighted cost surface representing the jamestown wastewater treatment plant, which had the lowest permitted average daily flow volume (0.4 million gallons^-day^) in this model. (JPG)

S1 Table The training point process dataset with measured triclosan concentrations. See S1 Metadata and text for variable explanations.
(CSV)

S2 Table The prediction point process dataset with predicted triclosan concentrations and differing discretization model contamination levels. See S1 Metadata and text for variable explanations.
(CSV)

S1 Metadata The metadata file for the training and prediction datasets (S1 and S2 Tables). (PDF)
