# Supplementary figures and images for: A Random Forest approach to predict the spatial distribution of sediment pollution in an estuarine system
Source: PLoS One. 2017 Jul 24;12(7):e0179473. doi: 10.1371/journal.pone.0179473 (PMC5524344; doi:10.1371/journal.pone.0179473)

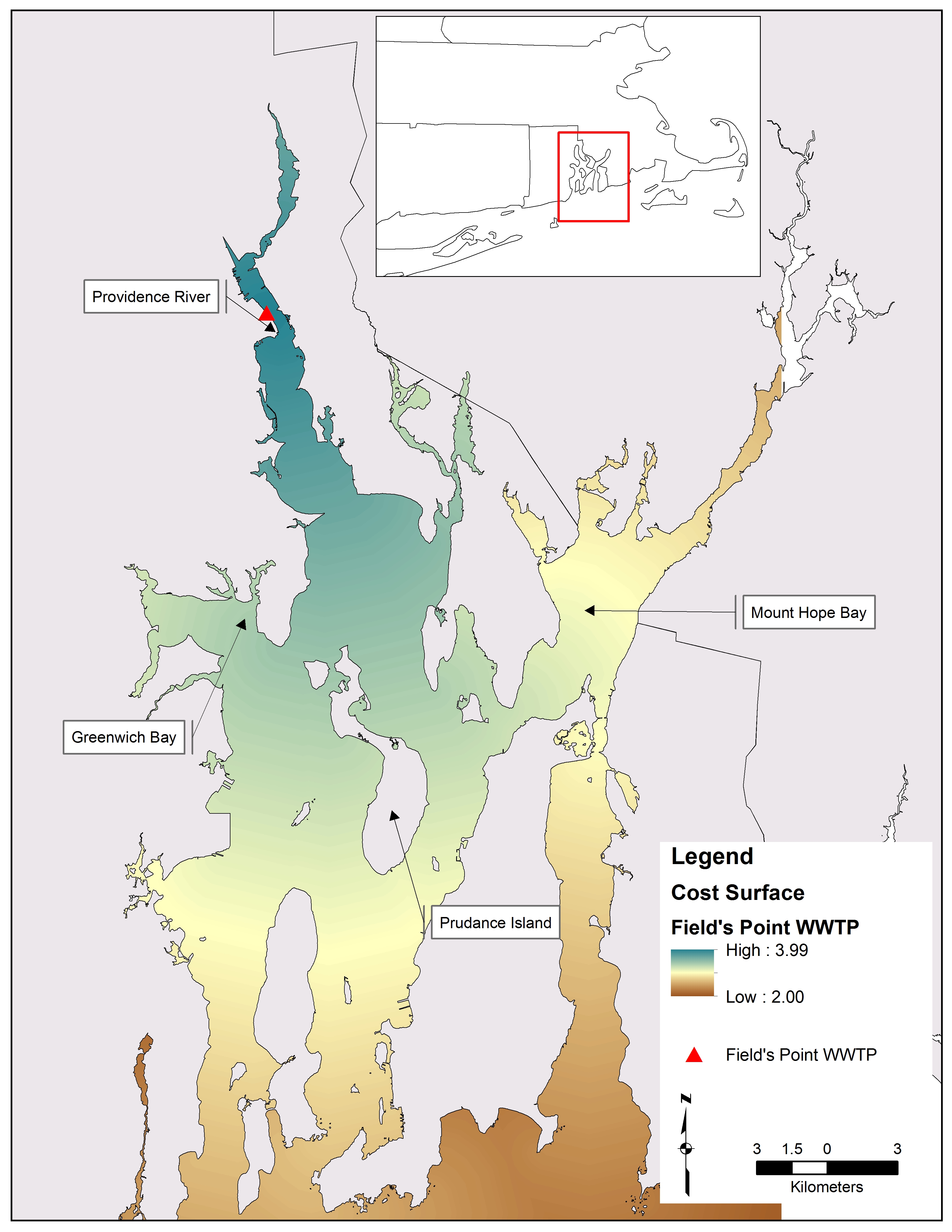

Supplement: S1 Fig — (JPG) [file pone.0179473.s001.jpg]

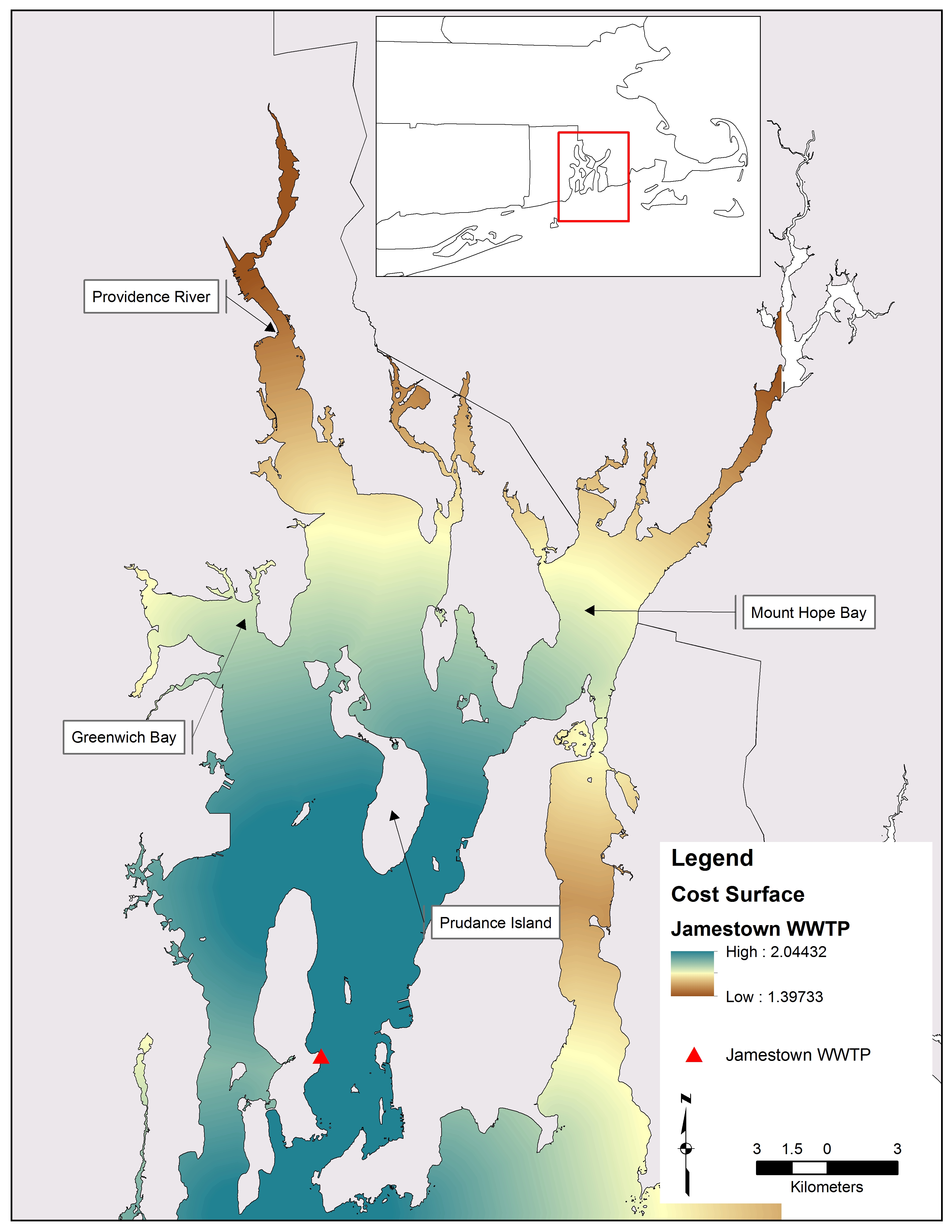

Supplement: S2 Fig — (JPG) [file pone.0179473.s002.jpg]
