# Supplementary material for: A Random Forest approach to predict the spatial distribution of sediment pollution in an estuarine system
Source: PLoS One. 2017 Jul 24;12(7):e0179473. doi: 10.1371/journal.pone.0179473 (PMC5524344; doi:10.1371/journal.pone.0179473)
Supplement: S1 Metadata — (PDF) [file pone.0179473.s005.pdf]

To Accompany Data for "A Random Forest Approach to Predict the Spatial Distribution of Triclosan in an Estuarine System"

Last updated: April 27, 2017

Sampling

Described at length in above mentioned manuscript by Walsh et al.

Data Sets: Below is the metadata for the data used in this study- a Random Forest (RF) model to predict the distribution of a model contaminant, triclosan (5-chloro-2-(2,4-dichlorophenoxy)phenol) (TCS), in Narragansett Bay, Rhode Island, USA. The RF explanatory variables were associated with TCS transport and fate (proxies) and direct and indirect environmental entry. Two data sets are presented. First, the training set that was used to develop the RF model. Second, the predictive data set that was used to explore the robustness of our model's predictions.

'training_20160504.csv': 58 obs. of 12 variables:

'prediction_20160504.csv': 446 obs. with 4 predicted TCS values/categories

---

Training Data

Field	Definition
TCS	Concentration Measured as ng/g of Dry Sediment
Northing	UTM Northing Coordinate (Zone 19)
Easting	UTM Easting Coordinate (Zone 19)
Depth	Bathymetry (ft)
Sand	Percent Sand
Mud	Percent Mud
WWTP_wt_Avg	Average Normalized Inverse Functional Distance Weighted by Permitted Discharge Volume
WWTP_Wt_Max	Maximum Normalized Inverse Functional Distance Weighted by Permitted Discharge Volume
WWTP_Wt_Min	Minimum Normalized Inverse Functional Distance Weighted by Permitted Discharge Volume
CSO_Avg_Dist	Average Functional Distance (m)
CSO_Min_Dist	Minimum Functional Distance (m)
CSO_Max_Dist	Maximum Functional Distance (m)
Carbon	Percent Total Organic Carbon

Prediction Data

**Field**	**Definition**
Northing	UTM Northing Coordinate (Zone 19)
Easting	UTM Easting Coordinate (Zone 19)
Depth	Bathymetry (ft)
Sand	Percent Sand
Mud	Percent Mud
Carbon	Percent Total Organic Carbon
CSO_Avg_Dist	Average Functional Distance (m)
CSO_Min_Dist	Minimum Functional Distance (m)
CSO_Max_Dist	Maximum Functional Distance (m)
WWTP_wt_Avg	Average Normalized Inverse Functional Distance Weighted by Permitted Discharge Volume
WWTP_Wt_Max	Maximum Normalized Inverse Functional Distance Weighted by Permitted Discharge Volume
WWTP_Wt_Min	Minimum Normalized Inverse Functional Distance Weighted by Permitted Discharge Volume
Predicted	RF Predicted TCS Concentration
TCSCat_a	Breaks Based on Equal Distribution of the Training TCS Concentration (Model A)
TCSCat_b	Breaks Based on 25% of Training Data into the Low and High Classes; 50% in the Medium Class (Model B)
TCSCat_c	Breaks Based on 12.5% of Training Data into the Low and High Classes (Model C)
